# Alzheimer Disease and Related Dementia Following Hormone-Modulating Therapy in Patients With Breast Cancer

**DOI:** 10.1001/jamanetworkopen.2024.22493

**Published:** 2024-07-16

**Authors:** Chao Cai, Kaowao Strickland, Sophia Knudsen, Sarah Beth Tucker, Chandana Sai Chidrala, Francesmary Modugno

**Affiliations:** 1 University of South Carolina, College of Pharmacy, Columbia; 2 South Carolina Revenue and Fiscal Affairs Office, Columbia, South Carolina; 3 University of South Carolina, Master of Health Information Technology, Columbia; 4 University of Pittsburgh, School of Medicine, Pittsburgh, Pennsylvania; 5 University of Pittsburgh School of Public Health, Pittsburgh, Pennsylvania; 6 Women’s Cancer Research Center, Magee-Womens Institute and Foundation and Hillman Cancer Center, Pittsburgh, Pennsylvania

## Abstract

**Importance:**

Hormone-modulating therapy (HMT) is a widely accepted treatment for hormone receptor–positive breast cancer, although its cognitive effects, including a potential link to Alzheimer disease and related dementias (ADRD), remain understudied.

**Objective:**

To investigate the association between HMT for breast cancer treatment and risk of developing ADRD in women aged 65 years or older.

**Design, Setting, and Participants:**

This cohort study used a comprehensive dataset from the Surveillance, Epidemiology, and End Results (SEER)–Medicare linked database to identify patients who did and did not receive HMT treatment within 3 years after the initial diagnosis of breast cancer and assessed their risk of developing ADRD in later life. Individuals with a preexisting diagnosis of ADRD or receiving HMT before the diagnosis of breast cancer were excluded. This study was performed from June 2022 through January 2024.

**Exposure:**

Receipt of HMT.

**Main Outcomes and Measures:**

Risk of ADRD associated with HMT; associations of risk with age, self-identified race, and HMT type. Risk was measured using hazard ratios (HRs) with 95% CIs and adjusted for potential confounders such as demographic, sociocultural, and clinical variables.

**Results:**

Among 18 808 women aged 65 years and older diagnosed with breast cancer between 2007 and 2009 (1266 Black [6.7%], 16 526 White [87.9%], 1016 other [5.4%]), 12 356 (65.7%) received HMT within 3 years after diagnosis, while 6452 (34.3%) did not. The most common age group in both samples was the 75 to 79 years age group (HMT, 2721 women [22.0%]; no HMT, 1469 women [22.8%]), and the majority of women in both groups self-identified as White (HMT, 10 904 women [88.3%]; no HMT, 5622 women [87.1%]). During an average of 12 years of follow-up, 2926 (23.7%) of HMT users and 1802 (27.9%) of non-HMT users developed ADRD. HMT was associated with a 7% lower relative risk of ADRD overall (HR, 0.93; 95% CI, 0.88-0.98; *P* = .005). The association decreased with age and varied by race. The reduction in ADRD risk associated with HMT was greatest for women aged 65 to 74 years who self-identified as Black (HR, 0.76; 95% CI, 0.62-0.92). This association decreased among women aged 75 years or older (HR, 0.81; 95% CI, 0.67-0.98). Women aged 65 to 74 years who self-identified as White had an 11% relative risk reduction (HR, 0.89; 95% CI, 0.81-0.97), but the association disappeared for women aged 75 years or older (HR, 0.96; 95% CI, 0.90-1.02). Other races showed no significant association between HMT and ADRD. Age- and race-based associations also varied by HMT type.

**Conclusions and Relevance:**

In this retrospective cohort study, hormone therapy was associated with protection against ADRD in women aged 65 years or older with newly diagnosed breast cancer; the decrease in risk was relatively greater for Black women and women under age 75 years, while the protective effect of HMT diminished with age and varied by race in women. When deciding to use HMT for breast cancer in women aged 65 years or more, clinicians should consider age, self-identified race, and HMT type in treatment decisions.

## Key Points

**Question:**

Does hormone-modulating therapy (HMT) for breast cancer treatment affect risk for Alzheimer disease and related dementias (ADRD)?

**Findings:**

This cohort study of 18 808 women aged 65 years or older with breast cancer showed HMT was associated with a 7% relative risk reduction in ADRD overall, with notable age and racial differences. Women aged 65 to 74 years who self-identified as Black experienced a 24% relative risk reduction compared with an 11% relative risk reduction in White patients; these associations diminished at age 75 years or older in White women but persisted in Black women.

**Meaning:**

These results suggest that the association of HMT and ADRD varies by age and race; when deciding on breast cancer treatment, therapy and individual factors must be considered.

## Teaser

This cohort study of US women aged 65 years and older diagnosed with breast cancer examines the association of hormone-modulating therapy with risk for Alzheimer disease and related dementias.

## Introduction

Breast cancer is the most commonly diagnosed cancer among women in the US,^1^ accounting for 31% of all new cancer cases.^1,2^ Age is a significant risk factor, with 83% of invasive breast cancers occurring in women aged 50 years and above.^2,3^ Although incidence rates of breast cancer have been increasing by about 0.5% per year since 2000, mortality rates have continued to decline.^2^ Thus, there are a growing number of breast cancer survivors in the US, including over 2.5 million breast cancer survivors over age 65, which accounts for more than half of women surviving the disease (4 million).^4^ This number is expected to increase by more than 7% by 2040, raising concerns about the impact of treatment-related complications^5^ among older adults.

In both men and women, about 10.8% of people aged 65 and older have Alzheimer disease (AD), with women accounting for almost two-thirds of the cases.^6,7^ Women aged 65 years and older with AD account for about 12% of the US population, while men represent a lower proportion at 9%.^6^ AD is currently the seventh-leading cause of death in the US and nearly 6 million people currently have AD.^8^ This number is expected to triple to 16 million by 2050.^9^ In addition to the growing number of persons with AD, the number of people with AD and related dementia (ADRD) has been increasing and is projected to continue rising in the coming years.^10,11^ The growing number of breast cancer survivors, especially among women aged 65 years and older, prompts significant concerns about increases in developing ADRD within this vulnerable group.

Breast cancer is commonly treated with surgery, chemotherapy, hormone-modulating therapy (HMT), biological therapy, or radiation therapy.^12,13,14,15^ About 83% of breast cancers are hormone receptor–positive and can be treated with hormonal therapy to block estrogen’s effects on cancer cell growth.^13,14,15^ Although long-term use of antiestrogen therapies has been associated with increased survival rates in breast cancer patients, there are also reports of decreased cognitive function, indicating potential increased risk of ADRD.^16,17^ The existing research on the association of hormone therapy on ADRD risk is sparse and shows inconsistent results.^18^ Some studies, including clinical trials and observational studies, suggest that HMT may lower the risk of cognitive conditions, such as mild cognitive impairment,^19^ ADRD,^20,21,22,23^ and neurodegenerative disease.^24^ Other studies suggest that HMT could be associated with an increased risk of ADRD^25,26,27,28,29^ or found no evidence to support the association.^30^ Research addressing ADRD risk following HMT in the breast cancer population is limited, leaving the precise relationship between HMT and later ADRD risk unclear.

This study used the Surveillance, Epidemiology, and End Results (SEER)–Medicare linked database, which combines SEER cancer registry data with Medicare claims,^31^ to evaluate the association between HMT use in newly diagnosed breast cancer and the risk of ADRD. Secondarily, we examined whether age or self-identified race changed any observed associations.

## Methods

### Data Source and Study Population

The study cohort, identified from the SEER-Medicare linked database, included demographic, sociocultural, and clinical information for individuals diagnosed with breast cancer. By establishing a linkage to Medicare claims, this database enables assessment of claims over a patient’s coverage period.^32,33^ This study was exempted by the University of South Carolina institutional review board from review and informed consent requirements due to retrospective analysis of deidentified data. This study followed the Strengthening the Reporting of Observational Studies in Epidemiology (STROBE) reporting guideline for reporting cohort studies.

**Figure 1.**
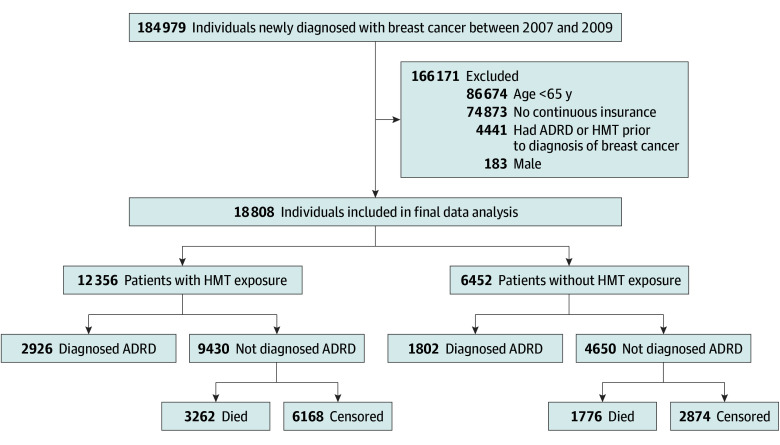
Description of Breast Cancer Population Diagnosed From 2007 to 2009 ADRD indicates Alzheimer disease and related dementias; HMT, hormone-modulating therapy. Patients were followed through December 31, 2019.

This study included women aged 65 years and older with newly diagnosed breast cancer from 2007 to 2009. Patients with preexisting diagnoses of ADRD or HMT use before the diagnosis and those without continuous insurance coverage were excluded (Figure 1). Inclusion criteria did not differentiate between breast cancer subtypes, tumor grades, or other specific cancer characteristics.

### Study Variables

HMT exposure was defined as initiating at least 1 HMT medication within 3 years after first breast cancer diagnosis. The HMT claims were determined by National Drug Code (NDC) and Healthcare Common Procedure Coding System (HCPCS) codes (eTable 1 in Supplement 1) for 3 types of HMT: (1) selective estrogen receptor modulators (SERMs), like tamoxifen and raloxifene; (2) aromatase inhibitors (AIs) such as anastrozole, letrozole, and exemestane; and (3) selective estrogen receptor degraders (SERDs) like fulvestrant.

The outcome was time to ADRD, identified using *International Classification of Diseases, Ninth Revision (ICD-9) *and *International Statistical Classification of Diseases and Related Health Problems, Tenth Revision (ICD-10)* codes (eTable 2 in Supplement 1). These diagnosis codes included ADRD subtypes ensuring that all ADRD patients were included.^33^ Patient-level demographic, sociocultural, medical, and treatment variables were evaluated as potential covariates.^24,34^ These included age at diagnosis, self-reported race, marital status, nursing home resident, poverty indicator, medical factors (chronic obstructive pulmonary disease, stroke, Charleston comorbidity index),^35^ and treatment factors (estrogen receptor [ER] status, progestogen receptor [PR] status, cancer grade, stage, other treatments including radiation, surgery, and chemotherapy)^36^ (eTable 3 and eTable 4 in Supplement 1). The cohort was followed from breast cancer diagnosis to the end of 2019, providing a minimum follow-up of 10 years.

### Statistical Analysis

Baseline demographic and clinical characteristics were summarized using unweighted and weighted descriptive statistics and evaluated by *t* tests or χ^2^ tests, as appropriate. The observational analog of the intention-to-treat (ITT) approach was used. Patients were assigned to HMT group based on their observed status as initiators of HMT, regardless of subsequent adherence or treatment discontinuation status during follow-up.^37,38^ A propensity score weighting approach was utilized to account for potential confounding and ensure balance among covariates between the HMT and non-HMT groups. A nonparsimonious logistic regression was used to estimate the propensity score, which is the probability of receiving HMT.^39,40,41^

Following the estimation of the propensity score, patients were weighted using a standardized mortality ratio (SMR) weighting approach,^42,43^ which was originally used in epidemiology to standardize risk of mortality. The traditional formula for SMR is observed outcomes/expected outcomes. This ratio adjusts for differences in confounding between the populations being compared. We adapted the concept of SMR within a propensity score framework to balance treatment groups. Specifically, the weights were created to estimate the average treatment effect on the treated patients (ATT). In the SMR weighting, we created a pseudosample in which HMT and non-HMT patients resemble the HMT population. This adaptation changes SMR from a simple ratio to a weighting mechanism where the HMT group is given a weight of 1, and the weight for the comparator (non-HMT) group is adjusted by the propensity score, calculated as (propensity score / [1 − propensity score]).^44^ This method, first proposed by Sato and Matsuyama in 2003,^45^ aims to simulate what the outcomes for the comparator group might have been had they received the treatment, thus providing a fair comparison and reducing bias in treatment effect estimates. Standardized differences were used to assess the differences in patient characteristics between groups. A standardized difference greater than 10% was considered an imbalance.^46^

Death is a common occurrence that could hinder the observation of ADRD outcomes in an older population. We addressed this by treating death as a competing risk. To address immortal time bias (the period between breast cancer diagnosis and initial HMT during which an outcome of ADRD cannot occur), the immortal time was counted toward the control group to prevent overestimating treatment effects (eFigure in Supplement 1).^47,48,49^

To evaluate the association between HMT and ADRD risk, 2 models were employed on the propensity score–weighted sample: model 1 treated death as competing risk and controlled for immortal time. Following this, a second model analyzed initiated HMT type on ADRD risk.

The nuanced association of HMT with the risk of developing ADRD was further investigated by conducting subgroup analyses across diverse age and self-identified racial groups to address potential health disparities. Racial groups were categorized as Black, White, and other based on self-identification. The other race group included American Indian or Alaskan Native, Asian or Pacific Islander, unspecified, and unknown. To examine how age and race modify the association of HMT with ADRD risk, our analysis separately introduced interactions of HMT with age and race into model 1. Additionally, to investigate how the type of HMT initiated affects susceptibility to ADRD across different age and racial groups, interactions of specific types of initiated HMT with age and race were separately introduced into model 2. To gain a thorough insight into how age and race are jointly associated with the risk of ADRD, we added both interactions between HMT with age and race into model 1. The same strategy was applied to model 2 when evaluating how age and race together may affect the association between the type of HMT initiated and ADRD risk. This methodology allowed for a nuanced understanding of HMT’s association, both overall and by specific types initiated, with ADRD risk across varied demographic groups. Statistical analyses were performed using SAS version 9.4 (SAS Institute Inc) and R version 4.2.2 (R Project for Statistical Computing). The statistical significance level was set at a 2-sided *P* value of .05. This study was performed from June 2022 through January 2024.

## Results

### Patient Characteristics

Of 184 979 patients newly diagnosed with breast cancer between 2007 and 2009, 18 808 women met inclusion and exclusion criteria and were included in the final data analysis. Among them, 12 356 women (65.7%) had HMT exposure within 3 years after diagnosis, while 6452 women (34.3%) were not exposed to HMT (Figure 1). The most common age group in both HMT use samples is the 75 to 79 years group (HMT, 2721 women [22.0%]; no HMT, 1469 women [22.8%]). Most women self-identified as White: the HMT cohort included 809 women (6.6%) identifying as Black, 10 904 (88.3%) as White, and 643 (5.2%) as other; the non-HMT cohort included 457 women (7.1%) identifying as Black, 5622 (87.1%) as White, and 373 (5.7%) as other. The mean (SD) age at diagnosis for the women in the HMT group was 75 (6) years vs 76 (7) years in the non-HMT group. Cases with ER-positive or PR-positive malignant neoplasms were more likely to receive HMT therapy within 3 years after diagnosis of breast cancer (8270 [66.9%] and 7070 [57.2%] vs 2444 [37.9%] and 2047 [31.7%] in the non-HMT cohort, respectively) (Table 1). The mean (SD) time to start HMT was 5.6 (4.7) months from diagnosis (range, 0-36 months). Among HMT users, 9409 (76.1%) initiated with AIs, 2915 (23.6%) initiated with SERMs, and only 32 patients (0.3%) initiated SERDs (eTable 5 in Supplement 1). Mean (SD) duration of HMT was 24 (9.5) months within 3 years following breast cancer diagnosis (range, 0-36 months).

**Table 1.** Baseline Characteristics of Women Aged 65 Years and Older Diagnosed With Breast Cancer in the SEER-Medicare Database, 2007-2009

Variables	Unweighted sample			Weighted sample		
	No. (%)		Standardized differences, %	No. (%)		Standardized differences, %
	HMT (n = 12 356)^a^	No HMT (n = 6452)		HMT (n = 12 356)^a^	No HMT (n = 8656)	
Age at diagnosis, y						
65-69	3311 (26.8)	1418 (22.0)	11.2	3311 (26.8)	2333 (27.0)	−0.4
70-74	3493 (28.3)	1529 (23.7)	10.4	3493 (28.3)	2436 (28.2)	0.3
75-79	2721 (22.0)	1469 (22.8)	−1.8	2721 (22.0)	1904 (22.0)	0.1
80-84	1852 (15.0)	1174 (18.2)	−8.6	1852 (15.0)	1294 (15.0)	0.1
85-89	769 (6.2)	644 (10.0)	−13.8	769 (6.2)	527 (6.1)	0.6
≥90	210 (1.7)	218 (3.4)	−10.7	210 (1.7)	160 (1.9)	−1.1
Race						
Black	809 (6.6)	457 (7.1)	−2.1	809 (6.6)	582 (6.7)	−0.7
White	10 904 (88.3)	5622 (87.1)	3.4	10 904 (88.3)	7533 (87.0)	3.7
Other^b^	643 (5.2)	373 (5.8)	−2.5	643 (5.2)	539 (6.2)	−4.4
Married	3912 (31.7)	1845 (28.6)	6.7	3912 (44.7)	3777 (43.6)	2.1
Poverty indicator^c^						
1	3213 (26.0)	1753 (27.2)	−2.6	3213 (26.0)	2161 (25.0)	2.4
2	3249 (26.3)	1646 (25.5)	1.8	3249 (26.3)	2169 (25.1)	2.8
3	3133 (25.4)	1639 (25.4)	−0.1	3133 (25.4)	2251 (26.0)	−1.5
4	1996 (16.2)	988 (15.3)	2.3	1996 (16.2)	1390 (16.1)	0.2
9	765 (6.2)	426 (6.6)	−1.7	765 (6.2)	683 (7.9)	−6.7
Nursing home resident	1050 (8.5)	748 (11.6)	−10.3	1050 (8.5)	717 (8.3)	0.8
History of chronic obstructive pulmonary disease	1549 (12.5)	870 (13.5)	−2.8	1549 (12.5)	1095 (12.7)	−0.4
History of stroke	1212 (9.8)	702 (10.9)	−3.5	1212 (9.8)	819 (9.5)	1.1
Charlson comorbidity index						
1	6693 (54.2)	3352 (52.0)	4.4	6693 (54.2)	4698 (54.3)	−0.2
2	4540 (36.7)	2508 (38.9)	−4.4	4540 (36.7)	3192 (36.9)	−0.3
3	780 (6.3)	440 (6.8)	−2	780 (6.3)	542 (6.3)	0.2
4	188 (1.5)	95 (1.5)	0.4	188 (1.5)	118 (1.4)	1.3
Tumor grade						
1	2968 (24.0)	1234 (19.1)	11.9	2968 (24.0)	2108 (24.4)	−0.8
2	5888 (47.7)	2099 (32.5)	31.2	5888 (47.7)	4039 (46.7)	2
3	2358 (19.1)	2042 (31.7)	−29.2	2358 (19.1)	1735 (20.1)	−2.4
Other	1142 (9.2)	1077 (16.7)	−22.3	1142 (9.2)	773 (8.9)	1.1
Stage						
In situ, localized, or regional	11 921 (96.5)	6270 (97.2)	−4	11 921 (96.5)	8497 (98.2)	−10
Distant	319 (2.6)	70 (1.1)	11.2	319 (2.6)	99 (1.1)	10
Other	116 (0.9)	112 (1.7)	−6.9	116 (0.9)	60 (0.7)	2.7
Tumor ER-positive	8270 (66.9)	2444 (37.9)	60.8	8270 (66.9)	8059 (66.3)	1.3
Tumor PR-positive	7070 (57.2)	2047 (31.7)	53.1	7070 (57.2)	7019 (57.7)	−1.1
No. of other treatments (radiation, chemotherapy, and surgery)						
0	7225 (58.4)	3421 (53.0)	11.0	7225 (58.5)	5058 (58.4)	0.1
1	3454 (28.0)	1554 (24.1)	8.8	3454 (28.0)	2430 (28.1)	−0.3
≥2	594 (4.8)	242 (3.8)	5.2	594 (4.8)	406 (4.7)	0.6
Chemotherapy	3041 (24.6)	1381 (21.4)	7.6	3041 (24.6)	2130 (24.6)	0
Surgery	1883 (15.2)	877 (13.6)	4.7	1883 (15.2)	1394 (16.1)	−2.4
Radiation	10 991 (89.0)	4997 (77.4)	31.1	10 991 (89.0)	7609 (87.9)	3.3

Abbreviations: ER, estrogen receptor; HMT, hormone-modulating therapy; PR, progesterone receptor; SEER, Surveillance, Epidemiology, and End Results.

^a^ HMT group is defined as initiating HMT within 3 years after breast cancer diagnosis.

^b^ Other race group is defined as American Indian and Alaskan Native, Asian or Pacific Islander, unspecified, or unknown.

^c^ Categories for poverty indicator codes: 1 (0% to <5% poverty), 2 (5% to <10% poverty), 3 (10% to <20% poverty), 4 (20% poverty and above), and 9 (unknown or not applicable).

Propensity score weighting was used to address potential confounding effects and enhance comparability between the HMT and non-HMT groups.^42^ Before weighting, notable imbalances (ie, standardized mean differences above 10%) between the HMT and non-HMT groups were observed among demographic, sociocultural, and clinical variables. After propensity score weighting, equilibrium in baseline characteristics among all patients was achieved (Table 1). Thus, all subsequent analyses used propensity score weighting.

### ADRD Risk in HMT Users vs Non-HMT Users Among Breast Cancer Patients

Among 18 808 women with breast cancer, 2926 (23.7%) of HMT users and 1802 (27.9%) of non-HMT users developed ADRD by the end of the follow-up period. A total of 5038 women (26.8%) died during the follow-up period (3262 [26.4%] in HMT vs 1776 [27.5%] in non-HMT) (Figure 1). HMT use was associated with a statistically significant relative reduction in ADRD risk (HR, 0.93; 95% CI, 0.88-0.98; *P* = .005). When specifically comparing the type of HMT initiated with non-HMT, the hazard ratios for initiating AI and SERM were found to be statistically significant (AI: HR, 0.93; 95% CI, 0.88-0.99; *P* = .02; SERM: HR, 0.89; 95% CI, 0.81-0.96; *P* = .005). Results for initiating SERD were not significant (HR, 0.37; 95% CI, 0.13-1.05; *P* = .06) (Table 2).

**Table 2.** Association of ADRD Risk With HMT Among Women Aged 65 Years and Older Diagnosed With Breast Cancer, 2007-2009

Model	Propensity score weighted sample		
	Comparison	HR (95% CI)	*P* value
1 (Controlled competing risk and immortal time)	HMT vs non-HMT	0.93 (0.88-0.98)	.005
2 (Controlled competing risk and immortal time compared by type of HMT initiated)	AI vs non-HMT	0.93 (0.88-0.99)	.02
	SERM vs non-HMT	0.89 (0.81-0.96)	.005
	SERD vs non-HMT	0.37 (0.13-1.05)	.06

Abbreviations: ADRD, Alzheimer disease and related dementias; AI, aromatase inhibitor; HMT, hormone-modulating therapy; HR, hazard ratio; SERD, selective estrogen receptor degraders; SERM, selective estrogen receptor modulator.

### Subgroup Analyses

**Figure 2.**
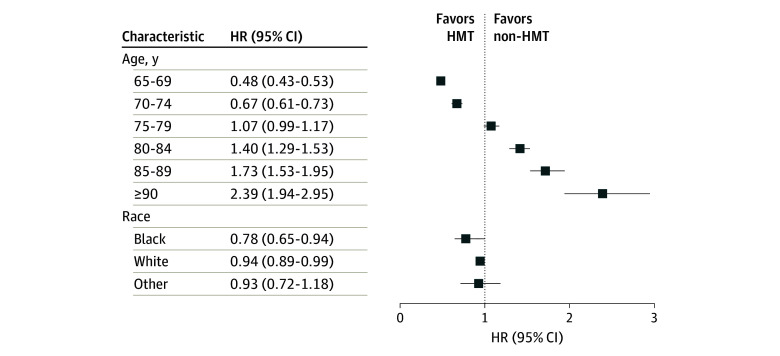
ADRD Risk Associated With HMT Stratified by Age and Self-Identified Race Among Women Aged 65 Years and Older Diagnosed With Breast Cancer, 2007-2009 ADRD, Alzheimer disease and related dementias; HMT, hormone-modulating therapy; HR, hazard ratio.

Subgroup analyses revealed the age-modified association of HMT and ADRD risk, with HRs varying significantly with age (interaction of HMT and age group, *P* < .001) (Figure 2). Reduced risk was most pronounced in the 65-to-69-year-old group (HR, 0.48; 95% CI, 0.43-0.53), and this association diminished with increasing age. Notably, when age reached 80 years, HMT transitioned into a positive association with ADRD risk (HR, 1.40; 95% CI, 1.29-1.53), and the risk association escalated with advancing age, reaching an HR of 2.39 (95% CI, 1.94-2.95) in women aged 90 years or more. Racial differences were also evident. Women who self-identified as Black experienced greater reductions in ADRD risk associated with HMT (Black: HR, 0.78; 95% CI, 0.65-0.94; White: HR, 0.94; 95% CI, 0.89-0.99; other: HR, 0.93; 95% CI, 0.72-1.18; interaction of HMT and race, *P* < .001) (Figure 2).

Considering the age-modified association of HMT and ADRD, we divided age into 2 groups (65 to 74 years and 75 years and older) to better understand the joint association of age and race with ADRD risk (Table 3). For Black women aged 65 to 74 years, HMT use was associated with significantly reduced risk of ADRD (HR, 0.76; 95% CI, 0.62-0.92), with AI initiation showing a slightly greater effect size (HR, 0.73; 95% CI, 0.59-0.91) than SERM (HR, 0.80; 95% CI, 0.57-1.11), although results for SERM were not significant. In Black women aged 75 years and above, the protective association persisted but the effect size was less pronounced (HMT vs non-HMT: HR, 0.81; 95% CI, 0.67-0.98). White women in the 65 to 74 years age group also appeared to benefit from HMT, showing a significant inverse association with ADRD risk (HR, 0.89; 95% CI, 0.81-0.97), especially with initiated SERM type (HR, 0.81; 95% CI, 0.70-0.94). However, among White women aged 75 years and above, HMT had no significant association with ADRD risk (HR, 0.96; 95% CI, 0.90-1.02). For women of other races, HMT did not significantly change the ADRD risk association in either age group. The age-race interactions for HMT overall and by subtypes were all statistically significant (all with *P* values < .001).

**Table 3.** Association of ADRD Risk With HMT by Type, Age, and Self-Identified Race Among Women Aged 65 Years and Older Diagnosed With Breast Cancer, 2007-2009

Characteristics	Propensity score weighted sample, HR (95% CI)^a^^,^^b^		
	HMT vs non-HMT	AI vs non-HMT	SERM vs non-HMT
Black			
65-74 y	0.76 (0.62-0.92)	0.73 (0.59-0.91)	0.80 (0.57-1.11)
≥75 y	0.81 (0.67-0.98)	0.78 (0.63-0.95)	0.91 (0.67-1.25)
White			
65-74 y	0.89 (0.81-0.97)	0.91 (0.82-1.00)	0.81 (0.70-0.94)
≥75 y	0.96 (0.90-1.02)	0.96 (0.90-1.03)	0.92 (0.83-1.03)
Other^c^			
65-74 y	0.87 (0.67-1.13)	0.88 (0.66-1.16)	0.81 (0.52-1.25)
≥75 y	0.93 (0.72-1.21)	0.93 (0.71-1.22)	0.92 (0.60-1.41)

Abbreviations: ADRD, Alzheimer disease and related dementias; AI, aromatase inhibitor; HMT, hormone-modulating therapy; SERD, selective estrogen receptor degraders; SERM, selective estrogen receptor modulator.

^a^ All interaction *P* values < .001.

^b^ SERD vs non-HMT was not included in the subgroup analysis due to small sample size.

^c^ Other race group is defined as American Indian or Alaskan Native, Asian or Pacific Islander, unspecified, or unknown.

## Discussion

HMT is a fundamental treatment for breast cancer patients with hormone positive disease.^24^ However, there remains concern regarding potential cognitive impairment following HMT initiation. The research on whether HMT is associated with the risk of ADRD has shown mixed results, leading to inconsistencies in the literature on this topic.

McCarthy et al^33^ verified the accuracy of ADRD diagnoses in Medicare claims, showing sensitivity of 31% to 57% and specificity of 92% to 98%. Positive predictive value ranged from 54% to 70%, and negative predictive value was 97%, confirming reliability in the diagnostic process used in Medicare claims data. Using one of the largest cohorts to date, we found that HMT use was associated with a significant 7% relative risk reduction in developing ADRD. These findings were consistent using various analytic approaches, including propensity score methods to address confounding, competing risk models to account for age-associated mortality risk, and models including immortal time to more accurately account for HMT exposure.^37^ Thus, these data provide confidence in the robustness of the association between HMT and ADRD.

Beyond examining the overall association of HMT and the risk of ADRD, our study highlights how age and race may affect ADRD risk. Specifically, we observed that HMT tends to be associated with greater protective benefits against ADRD in women aged 65 to 74 years, with this protective association weakening as age increases, notably beyond the age of 75 years. Moreover, we found that these age-related differences vary by self-identified racial groups. Younger Black women showed the most substantial protective associations between HMT and ADRD, with these associations diminishing yet remaining significant among Black women aged 75 years or older. Conversely, while White younger women also exhibited a reduced risk of ADRD associated with HMT, this association became insignificant after the age of 75 years. HMT was not associated with ADRD among women of other races. We further found that the specific type of HMT initiated, AI or SERM, also appears to influence the degree of ADRD risk associated with HMT. These findings emphasize the importance of considering age, race, and HMT type when evaluating the potential benefits of HMT on ADRD risk. The results highlight the critical need for personalized breast cancer treatment plans that are tailored to the individual characteristics of each patient, particularly given the significantly higher likelihood (2 to 3 times more) of Black women developing ADRD compared with their White counterparts.^50,51^

### Strengths and Limitations

The strengths of this study include the population-based dataset, large sample size, data representing actual clinical practice in the community, and detailed analyses using multiple statistical modeling approaches to address confounding, bias, and higher mortality due to competing events in this population. Despite these strengths, the study has limitations. The use of a Medicare population aged 65 years and older prohibits the generalizability of our findings to a broader age range. Moreover, our database lacks genetic information, such as the apolipoprotein E gene, and laboratory data associated with ADRD, including levels of β-amyloid and tau proteins in brain tissue.^52^ ER and PR status are strong predictors for breast cancer treatment. However, the role of these clinical characteristics as predictors for ADRD is less clear and is likely indirect. We treated ER and PR status as covariates and balanced them in the propensity score weighted sample. Further research is needed to understand the underlying mechanisms that might connect ER and PR status with ADRD risk, as mediated through HMT. While we examined the type of HMT, our analysis did not explore specific formulations of HMT, nor did we investigate how the duration of HMT affects the risk of ADRD. This study used ITT principles, so the duration of HMT therapy was not accounted for. While it is acknowledged that the duration of HMT can exceed 3 years, our study tracked HMT initiation only up to 3 years after breast cancer diagnosis to define exposure. Further investigation into longer identification windows for exposure, the timing of HMT initiation and the duration of therapy, is warranted to gain a more comprehensive understanding of their respective association with ADRD risk and shed light on optimal treatment regimens. Drug-related factors, including variations in drug types or formulations, the utilization of statins, antipsychotics, anxiolytics, hypnotics, antidepressants, and nonsteroidal anti-inflammatory drugs in the year preceding cohort entry were not assessed due to data availability. Despite using propensity scoring to control for confounding, potential confounders might remain concerning specific aspects of radiation, chemotherapy, and surgery—such as the intensity and duration of chemotherapy regimens and radiation effects on cognitive function. Future research should investigate these variables on HMT-ADRD relation.

## Conclusion

In this study, HMT was associated with protection against ADRD among women aged over 65 years with newly diagnosed breast cancer. Subgroup analyses further demonstrated the importance of considering age as an effect modifier in how HMT affects ADRD risk, as well as racial differences in the association across different ages and HMT types emphasizing the need for age-specific and race-specific strategies in addressing the risk factors and prevention efforts for ADRD associated with breast cancer treatment. Finally, while our results contribute valuable insights into the association of HMT with ADRD in the breast cancer population, further research is warranted to validate the observed associations in diverse populations and to elucidate the mechanisms underlying our observations.

## Notes

Supplement 1. **eTable 1.** Specific Codes for HMT Drugs **eTable 2.** Specific Codes for ADRD **eTable 3.** Specific Codes for Identifying Radiation and Surgery **eTable 4.** Specific Codes for Identifying Chemotherapy **eTable 5.** Frequency Table for HMT Users Among Women Aged 65 and Older Diagnosed With Breast Cancer in the SEER-Medicare Data, 2007-2009 **eFigure.** Problem of Immortal Time in HMT Group

Supplement 2.
Data Sharing Statement
