# Supplementary material for: Alzheimer Disease and Related Dementia Following Hormone-Modulating Therapy in Patients With Breast Cancer
Source: JAMA Netw Open. 2024 Jul 16;7(7):e2422493. doi: 10.1001/jamanetworkopen.2024.22493 (PMC11252894; doi:10.1001/jamanetworkopen.2024.22493)
Supplement: Supplement 1. — eTable 1. Specific Codes for HMT Drugs eTable 2. Specific Codes for ADRD eTable 3. Specific Codes for Identifying Radiation and Surgery eTable 4. Specific Codes for Identifying Chemotherapy eTable 5. Frequency Table for HMT Users Among Women Aged 65 and Older Diagnosed With Breast Cancer in the SEER-Medicare Data, 2007-2009 eFigure. Problem of Immortal Time in HMT Group [file jamanetwopen-e2422493-s001.pdf]

Supplemental Online Content

Cai C, Strickland K, Knudsen S, Tucker SB, Chidrala CS, Modugno F. Alzheimer disease and related dementia following hormone modulating therapy in patients with breast cancer. *JAMA Netw Open.* 2024;7(7):e2422493. doi:10.1001/jamanetworkopen.2024.22493

eTable 1. Specific Codes for HMT Drugs

eTable 2. Specific Codes for ADRD

eTable 3. Specific Codes for Identifying Radiation and Surgery

eTable 4. Specific Codes for Identifying Chemotherapy

eTable 5. Frequency Table for HMT Users Among Women Aged 65 and Older Diagnosed With Breast Cancer in the SEER-Medicare Data, 2007-2009

eFigure. Problem of Immortal Time in HMT Group

This supplemental material has been provided by the authors to give readers additional information about their work.

© 2024 Cai C et al. *JAMA Network Open.*

Supplementary tables and figures

eTable 1: Specific codes for HMT drugs

HMT drug name	HCPCS/CPT	NDC-9 Product codes
Raloxifene Hydrochloride		00002-4165, 00002-4184, 00093-7290, 00179-0151, 00179-0212, 00179-0220, 00591-2367, 42291-0726, 43353-0253, 43598-0505, 49999-0458, 50228-0306, 50268-0694, 52343-0137, 55289-0266, 65162-0057, 65862-0709, 66993-0417, 66993-0661, 69189-0418, 69189-0678, 69189-2367, 70518-2994, 70518-3046, 71209-0082
Tamoxifen Citrate	S0187	00093-0782, 00093-0784, 00179-0224, 00179-1952, 00310-0600, 00310-0604, 00378-0144, 00378-0274, 00555-0446, 00555-0904, 00591-2232, 00591-2472, 00591-2473, 13632-0123, 50090-0485, 50090-0942, 50090-1998, 50090-2533, 51862-0446, 51862-0447, 51862-0449, 51862-0450, 51862-0682, 54569-3765, 54569-5716, 54868-3004, 59651-0299, 59651-0300, 60429-0909, 60429-0910, 60505-3035, 60505-3036, 63629-4413, 63739-0269, 68084-0924, 68084-0935, 68382-0826, 68382-0827, 70518-1881, 70518-2721, 70771-1184, 70771-1185, 71335-1424
Anastrozole	S0170	00054-0164, 00093-7536, 00115-1261, 00179-0068, 00310-0201, 00378-6034, 00781-5356, 00904-6195, 00904-6229, 16571-0421, 16729-0035, 21695-0990, 42043-0180, 42254-0161, 42291-0085, 42291-0105, 43063-0383, 50090-1193, 50090-1918, 50090-2005, 50090-2118, 50090-2453, 50268-0075, 51079-0323, 51655-0638, 51991-0620, 54569-6198, 54868-5000, 54868-6130, 55111-0647, 59651-0236, 60258-0866, 60429-0286, 60505-2985, 60687-0112, 60763-0376, 62175-0710, 62559-0670, 62756-0250, 63187-0080, 63323-0129, 63629-5269, 63672-0015, 65841-0743, 66336-0533, 66435-0415, 67877-0171, 68001-0155, 68071-1682, 68084-0448, 68382-0209, 68788-6774,<br>69117-0003, 69189-0035, 70518-2420, 70518-2484,<br>70934-0488, 76519-1224
Letrozole		00054-0269, 00078-0249, 00078-0909, 00078-0916,<br>00078-0923, 00093-7620, 00179-0169, 00378-2071, 00527-1712, 00603-4180, 16729-0034, 17856-0032, 24535-0801, 24724-0030, 42254-0243, 42291-0373, 42291-0374, 50268-0476, 51991-0759, 55111-0646, 57884-2021, 59651-0180, 60505-3255, 62175-0888, 62756-0511, 68084-0803, 68382-0363, 69117-0004, 69189-7620, 70518-1869, 70518-2020
Exemestane	S0156	00009-7663, 00054-0080, 00378-5001, 00832-0595, 44278-0025, 47781-0108, 51991-0005, 59762-2858, 60687-0132, 63629-2056, 65162-0240, 68382-0383, 69097-0316, 70771-1374, 71921-0190
Fulvestrant	J9395	00310-0720, 00591-5019, 00781-3079, 00781-3492, 16714-0118, 16729-0436, 25021-0462, 43598-0262, 63323-0715, 67457-0311, 68001-0424, 68001-0484, 68462-0317, 68842-0301, 70121-1463, 70534-0002, 70860-0211, 71288-0555, 71731-6121, 72603-0105

Source: https://seer.cancer.gov/oncologytoolbox/canmed/ndconc/

© 2024 Cai C et al. *JAMA Network Open*.

eTable 2: Specific codes for ADRD

Diagnosis	ICD-9	ICD-10
Dementia	290, 29420, 29411, 29410, 29282, 2912, 29421	
Alzheimer disease	3310	G300, G301, G308, G309

© 2024 Cai C et al. *JAMA Network Open*.

eTable 3: Specific codes for identifying radiation and surgery

	ICD-9-CM (Diagnosis)	ICD-9-PCS (Procedure)	HCPCS/CPT	Revenue Center Codes
Radiation	V58.0, V67.1	60.99, 92.2, 92.20, 92.21-92.29, 92.3, 92.30-92.39, 92.4, 92.41	0182T, 77750-77799, C1715, C1716, C1717, C1719, C2616, C2634-C2645, C2698, C2699, G0458, Q3001, 0073T, 0082T, 0182T,0197T, 55860, 55862, 55865,55875, 55876, 61793, 76872, 76873, 76965, 77261–79999, C1715, C1717, C2638-C2641, C2698, C2699, G0173, G0251, G0256, G0261, G0339, G0340, G0458, G6003, G6005, G6006, G6015, Q3001, S8049	0330, 0333, 0339, 0342
Surgery		17.42, 40.1, 40.2,40.3, 40.5, 40.53,40.59, 60.21, 60.29,60.2–60.6, 60.51-60.59, 60.61, 60.62,60.69	54690, 55810, 55812, 5815, 55821, 55831, 55840, 5842, 55845, 55866, 55899, S2900, 55873	

© 2024 Cai C et al. *JAMA Network Open*.

eTable 4: Specific codes for identifying chemotherapy

	ICD-9-CM (Diagnosis)	ICD-9-PCS (Procedure)	HCPCS/CPT	Revenue Center Codes	NDC-9 Codes
Chemotherapy	V58.1x	00.10, 17.70, 99.25, 99.28		0331, 0332, 0335	
Doxorubicin			C9415, J9000-J9002, Q2048-Q2050		00013-1116, 00013-1136, 00013-1146, 00013-1156, 00013-1176, 00013-1266, 00013-1286, 00015-3352, 00015-3353, 00069-0170, 00069-0171, 00069-3030, 00069-3031, 00069-3032, 00069-3033, 00069-3034, 00069-4004, 00069-4015, 00069-4026, 00069-4030, 00069-4031, 00069-4032, 00069-4033, 00069-4034, 00069-4037, 00143-9092, 00143-9093, 00143-9275, 00143-9277, 00143-9546, 00143-9547, 00143-9548, 00143-9549, 00338-0063, 00338-0067, 00338-0080, 00338-0086, 00409-0124, 00703-5040, 00703-5043, 00703-5046, 16714-0001, 16714-0742, 16714-0856, 25021-0207, 43598-0283, 43598-0541, 43598-0682, 43598-0683, 45963-0733, 47335-0049, 47335-0050, 47335-0082, 47335-0083, 47781-0256, 49315-0008, 49315-0009, 53150-0314, 53150-0315, 53150-0317, 53150-0320, 55390-0237, 55390-0238, 59676-0960, 59676-0966, 62756-0826, 62756-0827, 63323-0101, 63323-0883, 67457-0393, 67457-0394, 67457-0395, 67457-0396, 67457-0436, 67457-0478, 68001-0345, 68001-0492, 68001-0493, 68083-0248, 68083-0249, 68083-0250, 70121-1218, 70121-1219, 70710-1530, 70710-1531, 72603-0103, 72603-0200
Epirubicin			J9178, J9180		00009-5091, 00009-5093, 00115-1675, 00143-9202, 00143-9203, 00703-3067, 00703-3069, 25021-0203, 45963-0608, 53150-0247, 53150-0250, 59762-5091, 59762-5093, 59923-0701, 61703-0359, 66758-0042, 67457-0357, 67457-0358
Paclitaxel			C9127, C9431,		00069-0076, 00069-0078, 00069-0079, 00703-3213,

© 2024 Cai C et al. *JAMA Network Open*.

			J9264, J9265, J9267		00703-3216, 00703-3217, 00703-3218, 00703-4764, 00703-4766, 00703-4767, 00703-4768, 16714-0137, 25021-0213, 44567-0504, 44567-0505, 44567-0506, 45963-0613, 47781-0593, 47781-0594, 47781-0595, 51991-0937, 51991-0938, 55390-0114, 55390-0304, 55390-0314, 61703-0342, 63323-0763, 66758-0043, 67457-0434, 67457-0449, 67457-0471, 68083-0178, 68083-0179, 68083-0180, 68817-0134, 69539-0157, 69539-0158, 69539-0159, 70860-0200, 70860-0215, 72205-0061, 72205-0062, 72205-0063
Docetaxel			J9170, J9171		00069-9141, 00069-9142, 00069-9144, 00075-8001, 00075-8003, 00075-8004, 00075-8005, 00143-9204, 00143-9205, 00409-0201, 00409-0365, 00409-0366, 00409-0367, 00409-0368, 00409-0369, 00409-1732, 00409-4235, 00409-5068, 00409-7870, 00703-5720, 00703-5730, 00955-1020, 00955-1021, 00955-1022, 16714-0465, 16714-0500, 16729-0120, 16729-0228, 16729-0231, 16729-0267, 25021-0222, 25021-0245, 39822-2120, 39822-2180, 39822-2200, 42367-0121, 43066-0001, 43066-0006, 43066-0010, 43598-0258, 43598-0259, 43598-0389, 43598-0610, 43598-0611, 45963-0734, 45963-0765, 45963-0781, 45963-0790, 47335-0285, 47335-0323, 47335-0895, 47335-0939, 50742-0428, 50742-0431, 50742-0463, 55150-0378, 55150-0379, 55150-0380, 57884-3021, 63739-0932, 63739-0971, 66758-0050, 66758-0950, 67457-0531, 67457-0532, 67457-0781, 68083-0399, 68083-0400, 68083-0401, 69097-0369, 69097-0371, 70121-1221, 70121-1222, 70121-1223, 70700-0174, 70700-0175, 70700-0176, 71288-0143, 71288-0144, 71288-0150, 71288-0151, 72485-0214, 72485-0215, 72485-0216

© 2024 Cai C et al. *JAMA Network Open*.

Fluorouracil			J9190		00069-0169, 00069-0173, 00069-0174, 00069-0176, 00187-3204, 00378-4791, 00703-3015, 00703-3018, 00703-3019, 10139-0063, 16729-0276, 21695-0829, 25021-0215, 43547-0258, 50742-0423, 50742-0481, 50742-0482, 50742-0483, 51672-4063, 51672-4118, 51862-0362, 52549-4118, 54868-0951, 54868-6293, 63323-0117, 66530-0249, 66758-0044, 66758-0054, 68001-0266, 68083-0292, 68083-0293, 68682-0004, 68682-0085
Capecitabine			J8520, J8521		00004-1100, 00004-1101, 00054-0271, 00054-0272, 00093-7473, 00093-7474. 00179-0149, 00179-0195, 00378-2511, 00378-2512, 16714-0467, 16714-0468, 16729-0072, 16729-0073, 42291-0166, 42291-0167, 42291-0190, 42291-0191, 50268-0154, 51079-0510, 53808-0411, 54868-4143, 54868-5260, 55111-0496, 55111-0497, 59651-0204, 59651-0205, 59923-0721, 59923-0722, 60687-0149, 62756-0238, 62756-0239, 63759-3000, 63759-3001, 64980-0276, 64980-0277, 65162-0843, 65162-0844, 68001-0487, 68001-0488, 69097-0948, 69097-0949, 69539-0019, 69539-0020, 70756-0815, 70756-0816, 72205-0006, 72205-0007, 72485-0204, 72485-0205, 72606-0554, 72606-0555
Cyclophosphamide			C9420, C9421, J8530, J9070, J9080, J9090, J9091-J9097		00015-0502, 00015-0503, 00015-0504, 00015-0505, 00015-0506, 00054-0382, 00054-0383, 00054-4129, 00054-4130, 00781-3233, 00781-3244, 00781-3255, 10019-0935, 10019-0936, 10019-0937, 10019-0938, 10019-0939, 10019-0942, 10019-0943, 10019-0944, 10019-0945, 10019-0955, 10019-0956, 10019-0957, 10019-0982, 10019-0984, 10019-0988, 10019-0989, 10019-0990, 16714-0857, 16714-0858, 16714-0859, 43975-0307, 43975-0308, 50742-0519, 50742-0520, 54868-5005, 54868-5218,

© 2024 Cai C et al. *JAMA Network Open*.

					54879-0021, 54879-0022, 62559-0930, 62559-0931, 68001-0370, 68001-0371, 68001-0372, 68001-0442, 68001-0443, 68001-0444, 69097-0516, 69097-0517, 69189-0382, 69189-0383, 70121-1238, 70121-1239, 70121-1240, 70860-0218, 72603-0104, 72603-0326, 72603-0411
Carboplatin			J9045		00015-3210, 00015-3211, 00015-3212, 00015-3213, 00015-3214, 00015-3215, 00015-3216, 00703-3249, 00703-4239, 00703-4244, 00703-4246, 00703-4248, 16729-0295, 25021-0202, 47335-0150, 47335-0151, 47335-0284, 47781-0603, 47781-0604, 47781-0605, 47781-0606, 50742-0445, 50742-0446, 50742-0447, 50742-0448, 55150-0386, 57277-0105, 57277-0106, 57277-0107, 61703-0339, 61703-0360, 63323-0172, 66758-0047, 67457-0491, 67457-0492, 67457-0493, 67457-0494, 67457-0608, 68083-0190, 68083-0191, 68083-0192, 68083-0193, 69448-0005, 71288-0100
Cisplatin			C9418, J9060, J9062		00015-3070, 00015-3072, 00069-0081, 00069-0084, 00703-5747, 00703-5748, 16729-0288, 44567-0509, 44567-0510, 44567-0511, 44567-0530, 47781-0609, 47781-0610, 61126-0003, 61126-0004, 63323-0103, 67457-0424, 67457-0425, 68001-0283, 68083-0162, 68083-0163, 70860-0206
Vinorelbine			C9440, J9390		00008-0045, 00069-0099, 00069-0103, 00069-0205, 00703-4182, 00703-4183, 25021-0204, 45963-0607, 50742-0420, 50742-0427, 55390-0069, 55390-0070, 61703-0341, 64370-0532, 66758-0045, 67457-0431, 67457-0479, 67457-0481, 67457-0482
Gemcitabine			J9201, J9198		00002-7501, 00002-7502, 00069-3857, 00069-3858, 00069-3859, 00143-9394, 00143-9395, 00409-0181, 00409-0182, 00409-0183, 00409-0185, 00409-0186, 00409-0187, 00591-3562, 00591-3563, 00703-5775,

© 2024 Cai C et al. *JAMA Network Open*.

					00703-5778, 00781-3282, 00781-3283, 16714-0909, 16714-0930, 16729-0092, 16729-0117, 16729-0118, 16729-0391, 16729-0419, 16729-0423, 16729-0426, 23155-0213, 23155-0214, 23155-0483, 23155-0484, 23155-0528, 23155-0529, 25021-0208, 25021-0209, 25021-0234, 25021-0235, 25021-0239, 42236-0001, 42236-0002, 45963-0612, 45963-0619, 45963-0620, 45963-0623, 45963-0624, 45963-0636, 47335-0153, 47335-0154, 50742-0496, 50742-0497, 50742-0498, 55111-0686, 55111-0687, 55390-0391, 60505-6113, 60505-6114, 60505-6115, 62756-0008, 62756-0073, 62756-0102, 62756-0219, 62756-0321, 62756-0438, 62756-0533, 62756-0614, 62756-0746, 62756-0974, 63323-0102, 63323-0125, 63323-0126, 63759-3028, 63759-3029, 67457-0462, 67457-0463, 67457-0464, 67457-0616, 67457-0617, 67457-0618, 68001-0282, 68001-0342, 68001-0348, 68001-0350, 68001-0359, 68083-0148, 68083-0149, 69097-0313, 69097-0314, 70860-0204, 70860-0205, 71288-0113, 71288-0114, 71288-0117, 72485-0221, 72485-0222, 72485-0223
Ixabepilone			C9240, J9207		00015-1910, 00015-1911, 70020-1910, 70020-1911
Eribulin			C9280, J9179		62856-0389
Daunorubicin			C9024, C9424, J9150, J9151		00024-5858, 00143-9550, 00143-9551, 00703-5233, 10885-0001, 42658-0007, 42658-0019, 42658-0021, 55390-0108, 55390-0142, 55390-0805, 61958-0301, 68727-0745
Vinblastine			J9360		55390-0091, 63323-0278
Thiotepa			C9433, J9340		00143-9565, 25021-0246, 43598-0650, 53964-0001, 53964-0002, 55390-0030, 68083-0446, 69539-0123, 69539-0124, 70121-1631, 72205-0045, 72205-0046
Methotrexate			J8610, J9250, J9260		00054-4550, 00054-8550, 00069-0146, 00069-0147, 00069-0148, 00069-0149, 00069-0181, 00069-0204, 00143-9516, 00143-9517,

© 2024 Cai C et al. *JAMA Network Open.*

					00143-9518, 00143-9830, 00378-0014, 00555-0572, 00703-3671, 00703-3673, 00703-3675, 00703-3678, 00904-6012, 10139-0062, 16729-0277, 16729-0486, 21695-0111, 42291-0505, 42291-0594, 43063-0439, 49349-0314, 49349-0406, 50090-0294, 50090-2345, 50268-0527, 51079-0670, 51079-0670, 51285-0366, 51285-0367, 51285-0368, 51285-0369, 52652-2001, 54569-1818, 54868-0173, 54868-3826, 54868-4716, 55289-0924, 55390-0031, 55390-0032, 55390-0033, 55390-0034, 55390-0143, 59651-0182, 61703-0350, 61703-0351, 61703-0352, 61703-0408, 61786-0417, 63323-0121, 63323-0122, 63323-0123, 63629-1472, 66336-0338, 66758-0040, 66758-0041, 67253-0320, 67457-0221, 67457-0465, 67457-0466, 67457-0467, 67457-0480, 67457-0485, 67457-0486, 68382-0775, 69238-1423, 70518-0223, 70518-1251, 70518-1398, 70518-2711, 70771-1058, 71335-1772, 75840-0111
Vincristine			J9370, J9371, J9375, J9380		00703-4402, 00703-4412, 20536-0322, 61703-0309

© 2024 Cai C et al. *JAMA Network Open*.

eTable 5: Frequency table for HMT users (N = 12 356) among women aged 65 and older diagnosed with breast cancer in the SEER-Medicare data, 2007-2009.

HMT	No. (%)
Only one HMT	10732 (87)
> 1 HMT	1624 (13)
**HMT initiate type**	**No. (%)**
AI	9409 (76)
SERMS	2915 (24)
SERDS	32 (0.3)

© 2024 Cai C et al. *JAMA Network Open*.

eFigure. Problem of immortal time in HMT group. Immortal time refers to a period during which an outcome cannot occur. For the HMT group, this is the time from breast cancer diagnosis to start of HMT. To address this methodological challenge, immortal time in HMT was counted as untreated time.

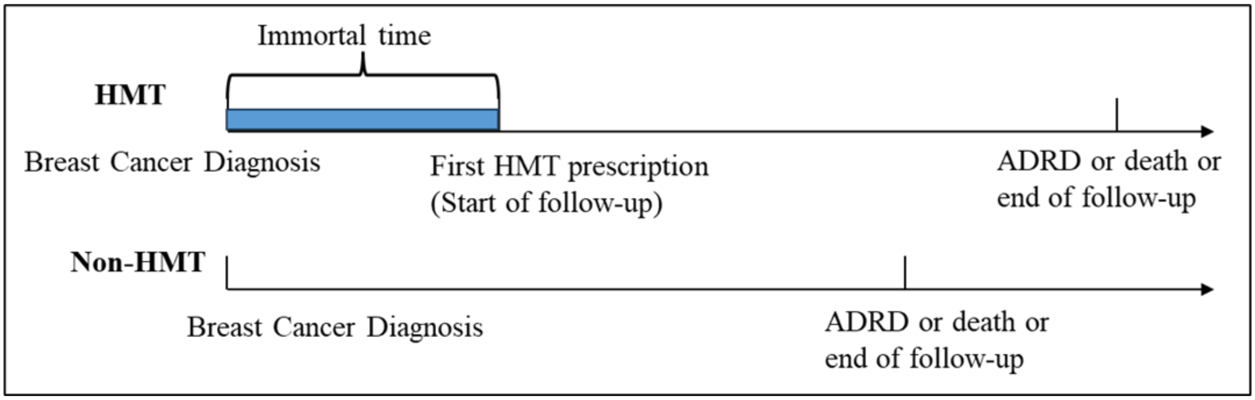

© 2024 Cai C et al. *JAMA Network Open*.
