# Supplementary material for: Alzheimer Disease and Related Dementia Following Hormone-Modulating Therapy in Patients With Breast Cancer
Source: JAMA Netw Open. 2024 Jul 16;7(7):e2422493. doi: 10.1001/jamanetworkopen.2024.22493 (PMC11252894; doi:10.1001/jamanetworkopen.2024.22493)
Supplement: Supplement 2. — Data Sharing Statement [file jamanetwopen-e2422493-s002.pdf]

Data Sharing Statement

Cai. Alzheimer Disease and Related Dementia Following Hormone Modulating Therapy in Patients with Breast Cancer. *JAMA Netw Open*. Published July 16, 2024.
doi:10.1001/jamanetworkopen.2024.22493

Data
Data available: No
